# Investigation of the viral and bacterial microbiota in intestinal samples from mink (*Neovison vison*) with pre-weaning diarrhea syndrome using next generation sequencing

**DOI:** 10.1371/journal.pone.0205890

**Published:** 2018-10-18

**Authors:** Julie Melsted Birch, Karin Ullman, Tina Struve, Jens Frederik Agger, Anne Sofie Hammer, Mikael Leijon, Henrik Elvang Jensen

**Affiliations:** 1 Department of Veterinary and Animal Sciences, Faculty of Health and Medical Sciences, University of Copenhagen, Frederiksberg C, Denmark; 2 Department of Microbiology, National Veterinary Institute, Uppsala, Sweden; 3 Kopenhagen Fur Diagnostics, Kopenhagen Fur, Glostrup, Denmark; Leibniz Institute for Zoo and Wildlife Research (IZW), GERMANY

## Abstract

Pre-weaning diarrhea (PWD) in mink kits is a common multifactorial syndrome on commercial mink farms. Several potential pathogens such as astroviruses, caliciviruses, *Escherichia coli* and *Staphylococcus delphini* have been studied, but the etiology of the syndrome seems complex. In pooled samples from 38 diarrheic and 42 non-diarrheic litters, each comprising of intestinal contents from 2–3 mink kits from the same litter, the bacterial populations were studied using Illumina Next Generation Sequencing technology and targeted 16S amplicon sequencing. In addition, we used deep sequencing to determine and compare the viral intestinal content in 31 healthy non-diarrheic and 30 diarrheic pooled samples (2–3 mink kits from the same litter per pool). The results showed high variations in composition of the bacterial species between the pools. Enterococci, staphylococci and streptococci dominated in both diarrheic and non-diarrheic pools. However, enterococci accounted for 70% of the reads in the diarrheic group compared to 50% in the non-diarrheic group and this increase was at the expense of staphylococci and streptococci which together accounted for 45% and 17% of the reads in the non-diarrheic and diarrheic group, respectively. Moreover, in the diarrheic pools there were more reads assigned to Clostridia, *Escherichia-Shigella* and *Enterobacter* compared to the non-diarrheic pools. The taxonomically categorized sequences from the virome showed that the most prevalent viruses in all pools were caliciviruses and mamastroviruses (almost exclusively type 10). However, the numbers of reads assigned to caliciviruses were almost 3 times higher in the diarrheic pools compared the non-diarrheic pools and *Sapporo*-like caliciviruses were more abundant than the *Norwalk*-like caliciviruses. The results from this study have contributed to the insight into the changes in the intestinal microbiota associated with the PWD syndrome of mink.

## Introduction

Pre-weaning diarrhea (PWD) affecting suckling mink kits is a well-known syndrome in commercial mink production and farmers usually refer to this as “sticky kits”, “wet kits” or “greasy kits”. In Denmark the syndrome was described more than 60 years ago [1] and normally affects kits from 1–4 weeks of age, in the suckling state of life [2–5]. It is characterized by diarrhea and a cutaneous exudation leading to a sticky appearance [2–5]. Outbreaks of PWD occur on mink farms with variable severity between years and the morbidity may range from affecting a few litters up to more than 30% [6,7]. The mortality is usually 1–2 kits per litter and the impact of an outbreak may have serious economic consequences for the farmers due to treatments, losses of animals and experience of decreased welfare as well. Presently, it is generally accepted that the syndrome is multifactorial and infectious agents together with environmental and genetic risk factors have been suggested as causes [2,6,7–9]. Associations between diarrhea in mink kits and different incriminated viruses and bacteria have been reported [10–18]. Many of these microorganisms have been identified in both healthy and diarrheic mink kits, which leave their role in PWD unclear. Generally, mink host many different viruses and studies have focused on astro-, calici-, rota- and coronaviruses in regard to the PWD syndrome [10–17]. Besides *Escherichia coli* (*E*. *coli*) *Staphylococcus spp*. have been incriminated in the PWD syndrome in mink [14,16,17–18]. Enteric colonization by *S*. *delphini* has besides in mink kits been reported in ferret kits with diarrhea and comparison to the “sticky kits” syndrome was proposed [19]. *S*. *delphini* has also been isolated from different extra intestinal sites in mink including the urinary tract and skin [20–21], and a study showed that mustelids like mink are natural hosts of *S*. *delphini* group A [20].

Studying bacterial etiologic candidates for PWD indeed require knowledge about the normal intestinal microflora in mink at different ages. Research on the intestinal microflora in mink is sparse and mainly based on culture studies [22–24]. Older conventional culture based studies showed that the total count of bacteria in colon of adult and juvenile mink is low compared to other species like ruminants or humans and consist of approximately equal counts of aerobic and anaerobic bacterial species which is different from other animal species, where 90% or more consist of anaerobic bacteria [22–23,25–26]. However, some intestinal bacteria are not cultivable which is a drawback when interpreting these results, and recent studies using next generation sequencing (NGS) have shown that the adult mink microbiota is dominated by Firmicutes class Clostridiales although large inter-individual differences were demonstrated [27–28]. Adult mink also have a low amount of bacteroides compared to other species [23,26–29]. Mink are characterized by having a simple intestinal tract with a rapid passage time (3–4 hours) which might be even shorter in suckling kits [23,30]. A study showed that the fecal aerobic microflora in healthy mink kits were dominated by hemolytic staphylococci, *E*.*coli* and enterococci [24]. The employment of HTS (High-throughput Sequencing) techniques with bioinformatics data analysis allow more complex infection biology issues to be studied [31] and has for example been applied in the study of the enteric disease complex NNPD (New Neonatal Porcine Diarrhea) [32].

In the present study we performed NGS to determine the mink kit intestinal virome by identification of sequence reads using Basic Local Alignment Search Tool (BLASTx) [33]. In addition, targeted 16S rRNA amplicon sequencing for determination of the composition of the gut bacterial microbiota in intestinal contents from mink kits with PWD and from healthy kits were carried out. It was our aim to characterize and compare the virome and bacterial microbiota in diarrheic and non-diarrheic mink kits to examine potential viral and bacterial involvement in the PWD syndrome in mink. Although NGS techniques have been used to examine the bacterial microbiota in adult mink, this is the first time NGS techniques are applied in the investigation of the etiology of PWD in mink.

## Material and methods

### Sample collection

In the pre-weaning period from May 11 to June 2, 2015 the investigation team visited 30 Danish commercial mink farms with either high or low frequency of PWD. From each farm 2–4 litters with and without clinical signs of PWD were conveniently sampled. Clinical signs included cutaneous exudation, exudate covering the nails, red swollen anus, perineal soiling and dehydration (dry wrinkled skin). From the litters, 2–3 mink kits were euthanized by injection of pentobarbital. The post-mortem examinations were conducted immediately after euthanization at the farm and the consistency and color of the intestinal contents in the aboral part of the gastrointestinal tract or at post mortem defecation was assessed. The gut was evacuated in its whole length and the contents were squeezed out into a cryo tube and immediately placed in a cool box for maximum 6 hours and subsequently frozen at -20°C. The samples were collectively transported on dry ice to the National Veterinary Institute (SVA) Uppsala, Sweden, where they were stored at -80°C until preparation. The definition of litters with diarrhea and litters without diarrhea was based on consistency of the feces/caudal intestinal contents in combination with external clinical signs (S1 Fig and S1 Table). Some of the mink farmers had initiated antimicrobial feed medication of the whole herd in the nursing period, thus 18 females had received antimicrobials within one week of sampling, but none of the mink kits had received any medication. In total 7 litters were excluded from the study because the litters had received solid feed or the disease status was impossible to determine due to improper recordings, and two litters were excluded because they only contained one kit. In total 39 diarrheic litters and 44 non-diarrheic litters were included in the study. The animals used in this project were solely euthanized for collection of their tissues/organs and thereby in accordance with the Danish Order regarding animal experimentation §2, article1. Therefore, approval from ethics committees was not required. Euthanasia was carried out humanely by injection of an overdose of pentobarbital by an authorized veterinarian and in accordance with the Danish Order regarding slaughter and euthanasia of animals.

### Sample preparation

After thawing, approximately 80μl of feces was mixed with 720 μl 1 x TURBO DNase Buffer (Thermo Fisher Scientific) and homogenized by vortexing for 30 sec. The suspensions were frozen once on dry ice before thawing and vortexed again. Pools of feces from mink kits from the same litter were generated in the way that for litters with 3 samples 400 μl from each sample were pooled to a total amount of 1200 μl and from litters with only two samples 600 μl from each sample were pooled to a total amount of 1200 μl. The pooled samples were centrifuged at 12000 x g for 5 min at 4°C which gave approximately 250 μl pellet and 950 μl supernatant. The supernatants were removed and stored at -80°C until used for extraction of viral RNA and the pellets were used for DNA extraction.

### 16S rRNA amplicon sequencing

#### DNA isolation

Total DNA was extracted from the pellet with the PowerLyzer PowerSoil DNA Isolation Kit (MO BIO laboratories, QIAGEN) according to the manufacturer´s protocol with minor modifications. Briefly, 250 μl pellet from the pooled samples was re-suspended in 750 μl Bead Solution and loaded into a Glass Bead Tube for homogenization. After adding the cell lysis solution (Solution C1) the samples were incubated for 10 min at 98°C. Homogenization was performed on a FastPrep-24 Sample Preparation System (MP Biomedicals) with the setting 6.5 m/s, for 2 times 60 sec. The following centrifugation was extended to 2 min. After incubation and centrifugation with Solution C3, 700 μl of the supernatant was transferred to a clean Collection Tube. DNA was eluted with 80 μl of the elution buffer heated to 65°C and allowed to work on the silica membrane for 2 min before centrifugation. The extracted DNA was stored at -20°C until continuing with the library preparation.

#### 16S library preparation

Library preparation for sequencing of the variable V3 and V4 regions of the 16S ribosomal RNA gene was done according to the protocol 16S Metagenomic Sequencing Library Preparation (Rev.B) recommended and applied for the Illumina MiSeq System (Illumina Inc, San Diego). The amplicon libraries were combined at equimolar concentrations, spiked with 5% PhiX control, denatured and loaded on a MiSeq flow cell and sequenced with a 600 cycles reagent kit v3 (Illumina Inc, San Diego) in paired-end sequencing runs.

### Virome sequencing

#### Virus enrichment and RNA extraction

The supernatant from the pooled sample preparation was filtered through a Millex-HPF HV 0.45 μm filter (Merck Millipore) by use of a 2-ml syringe to remove bacterial and host cells. In total 180 μl of the filtrate was treated with 20 μl Turbo DNase (Ambion) and 2 μl RNase ONE Ribonuclease (Promega) at 37°C for 30 min to degrade nucleic acids not protected in viral capsids. Nuclease digestion was stopped by adding three volumes (600 μl) of TRI Reagent LS (Sigma-Aldrich) to each sample and mixed for 5 min. An equal amount of ethanol (99,5%) was added and mixed thoroughly before the mixture was transferred into a Zymo-Spin column placed in a collection tube (Direct-zol RNA MiniPrep Kit, Zymo Research). The columns were centrifuged at 16000 x g for 30 sec. after which they were washed two times with 400 μl of PreWash solution each time followed by 30 sec. of centrifugation. Then 700 μl of RNA Wash Buffer was added to the columns followed by centrifugation for 2 min before they were transferred to RNase free Eppendorf tubes. RNA was eluted from the column matrix by adding 30 μl of DNase/RNase free water and centrifugation at 16,000 x g for 30 sec.

#### Synthesis of double-stranded cDNA

The concentration of RNA in the samples was measured with QUBIT RNA HS Kit in a Qubit 2.0 Flourometer (Invitrogen Thermo Fisher Scientific)). Using the SuperScript IV First-Strand Synthesis System (Invitrogen, Thermo Fisher Scientific) cDNA was synthesized with random hexamer on a Bio-Rad S1000 Thermal cycler according to the manufacturer’s instructions. RNase H was added to each well and incubated for 20 min at 37°C to destroy the template RNA. Ds-cDNA was synthesized by adding 0.5 μl of 3’-5’ exo- Klenow DNA polymerase (Klenow Fragment, New England BioLabs) and incubating for 60 min at 37°C followed by a 10-min enzyme inactivation step at 75°C. The concentration of ds-cDNA was measured with QUBIT dsDNA HS Kit (Invitrogen, Thermo Fisher Scientific) and normalized to 0.2 ng/μl for further library preparation.

#### Library preparation and sequencing

Libraries with dual indexing for each sample were generated with Nextera XT DNA Library Prep Kit (Illumina Inc, San Diego) according to the manufacturer´s instructions with the minor modifications that half the volumes were used for all reagents in the tagmentation and amplification steps. The libraries were cleaned up with AMPure XP beads (Beckman Coulter) and the size distribution and the concentration of each library were estimated using a High Sensitivity DNA Chip in an Agilent 2100 Bioanalyzer Instrument (Agilent). The libraries were diluted in Resuspension Buffer (Illumina) to 2 nM and pooled. After denaturation with 0.2 M NaOH, the pool of libraries was diluted in Hybridization Buffer (Illumina) to 10pM and combined with 1% denatured PhiX Control before sequencing on the Illumina MiSeq platform with MiSeq 600 Cycles Reagent Kit v3.

### Bioinformatics and data analysis

MiSeq paired-end reads originating from RNA-preparations for virus detection of the pooled feces samples were acquired as described above. The total number of pools generating sufficient data (a lower limit of 95 000 reads were employed) for further analysis were 30 and 31 for diarrheic and non-diarrheic pools, respectively. The total number of reads taxonomically assigned to viruses from diarrheic and non-diarrheic pools were 1.50 and 0.90 million, respectively, after scaling against the total numbers of reads in each category as implemented in MEGAN6 [34]. The reads were trimmed using CLC genomics workbench 10.1.1 (Qiagen Aarhus A/S). The sequence reads were then aligned to the ncbi nr database using the blastx algorithm as implemented in DIAMOND version 08.10 [35] with the e-value cutoff set to 10^−5^. The blast tables generated by DIAMOND were imported to MEGAN6 [34] for further analysis and taxonomic classification. For analysis of the bacterial content of the pooled samples 16S rRNA amplicon sequencing was carried out. The V3 and V4 regions were amplified producing a 459 bp amplicon. The MiSeq amplicon sequencing data were analyzed with the 16S amplicon data dedicated pipeline in the microbial genomics plugin module (version 2.5.1) of the CLC genomics workbench (version 10.1.1). The reads were trimmed based on quality parameters and samples with less than 40 000 reads were excluded. After this procedure 38 and 42 pools remained in the diarrheic and non-diarrheic groups, respectively, for further analysis. The Paired end reads were merged and verified to be of identical length before operational taxonomical unit (OTU) clustering using the SILVA OTU 16S database v. 123 with 97% as required similarity for assignment [36].

## Results

### Microbiota

**Fig 1:**
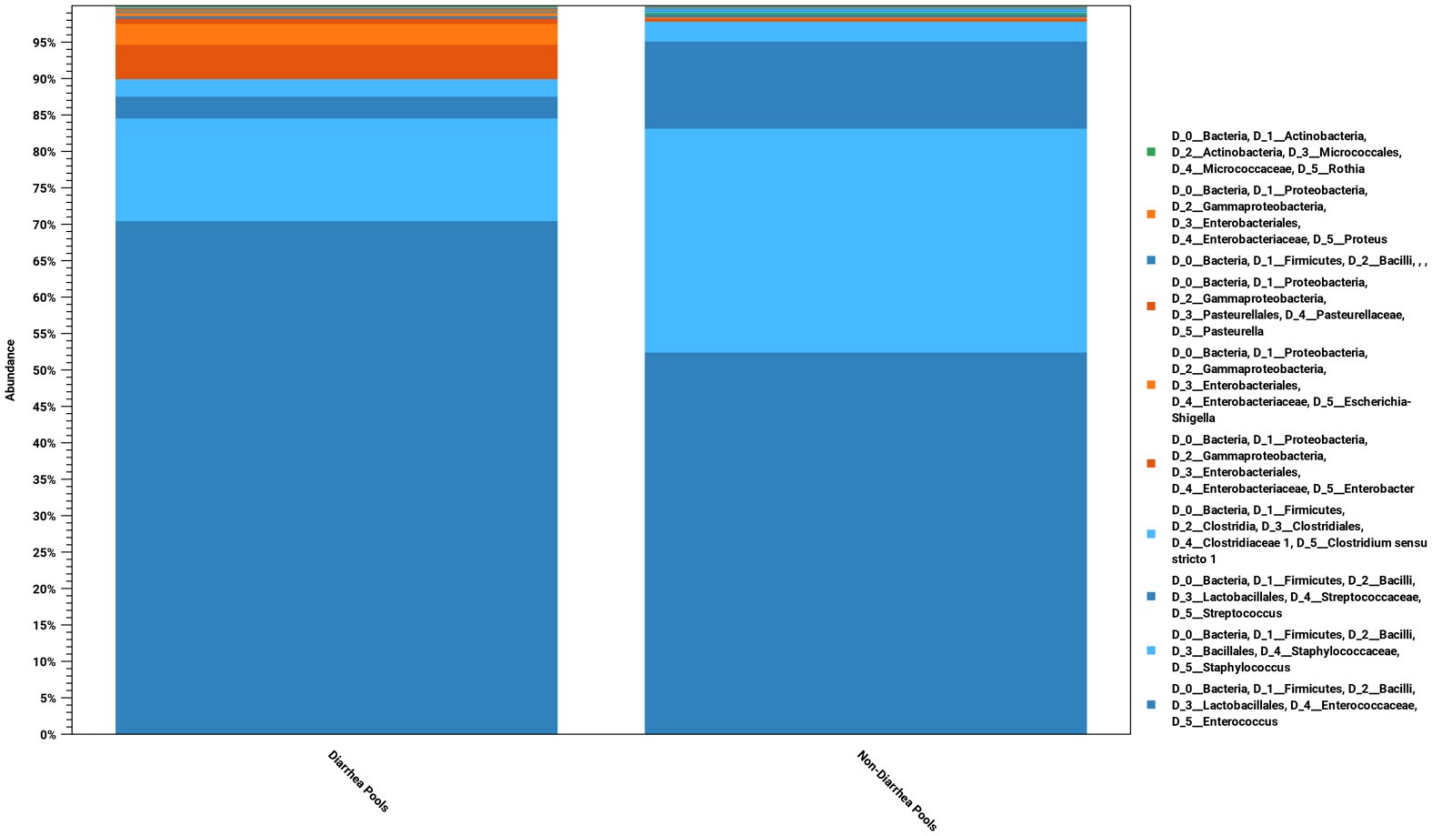
The bacterial populations derived from 16S rRNA sequence classification for the sum of all mink kit feces pools grouped as diarrheic and non-diarrheic. Of the 83 pools included in the study, 80 (38 diarrheic and 42 non-diarrheic) passed the set low coverage limit of 40 000 reads and contribute to the data shown. Two of the diarrhea pools and two of the non-diarrhea pools were run in duplicate and in total 84 pool samples are included. The populations are displayed aggregated and colored at the taxonomic genus and phylum levels, respectively.

In Fig 1 all the OTU-assigned 16S amplicons are grouped according to diarrheic/non-diarrheic status based on post-mortem examination. The bacterial populations are clearly different in the two groups. Generally firmicutes with bacillales and lactobacillales dominated in both groups with the most prevalent genera being enterococci (dark blue bottom), staphylococci (light blue bottom) and streptococci (dark blue top) (Note that in Fig 1 the aggregation of OTUs is on taxonomic genus level while the coloring is based on the phyla. Different shades of the colors are used when genera of the same phylum appear next to each other in the bars. The order of appearance of from bottom to top in the bars is determined by the prevalence and is given in the legend of the figure.). However, for the reads coming from diarrheic pools, there were also a significant number of reads assigned to Clostridia (light blue top), *Escherichia-Shigella* (orange) and *Enterobacter* (red).

**Fig 2:**
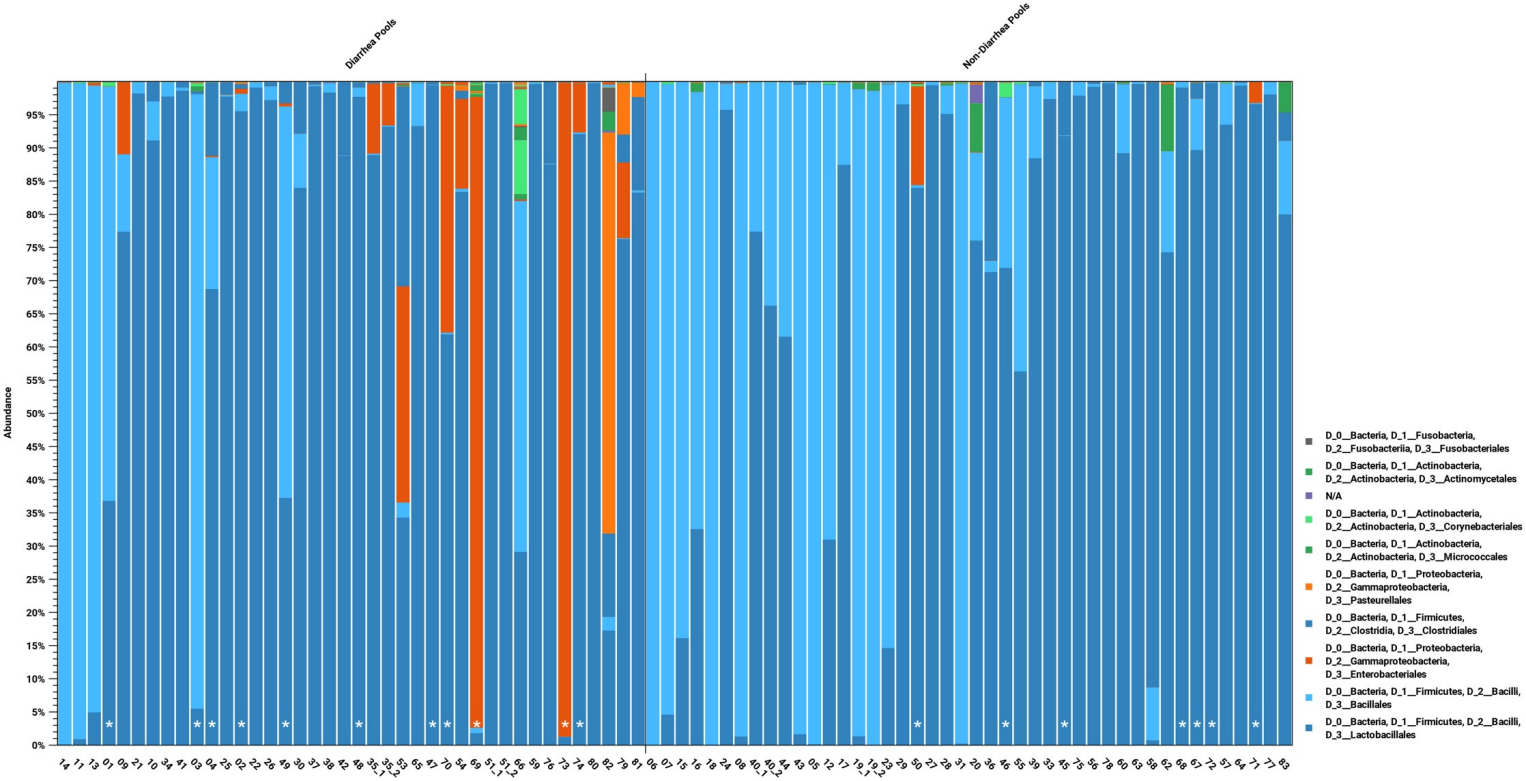
Individual bacterial populations derived from 16S rRNA sequence classification for each of the 84 mink kit feces pool samples grouped as diarrheic and non-diarrheic and ordered according to litter age. Two each of the diarrheic pools and non-diarrheic pools were run in duplicate. The stars (*) indicate pools from litters where the female was treated with the antibiotic amoxicillin. The populations are displayed aggregated and colored at the taxonomic order and phylum levels, respectively. The duplicates are indicated. The litter age of the pools is not show in this figure.

**Fig 3:**
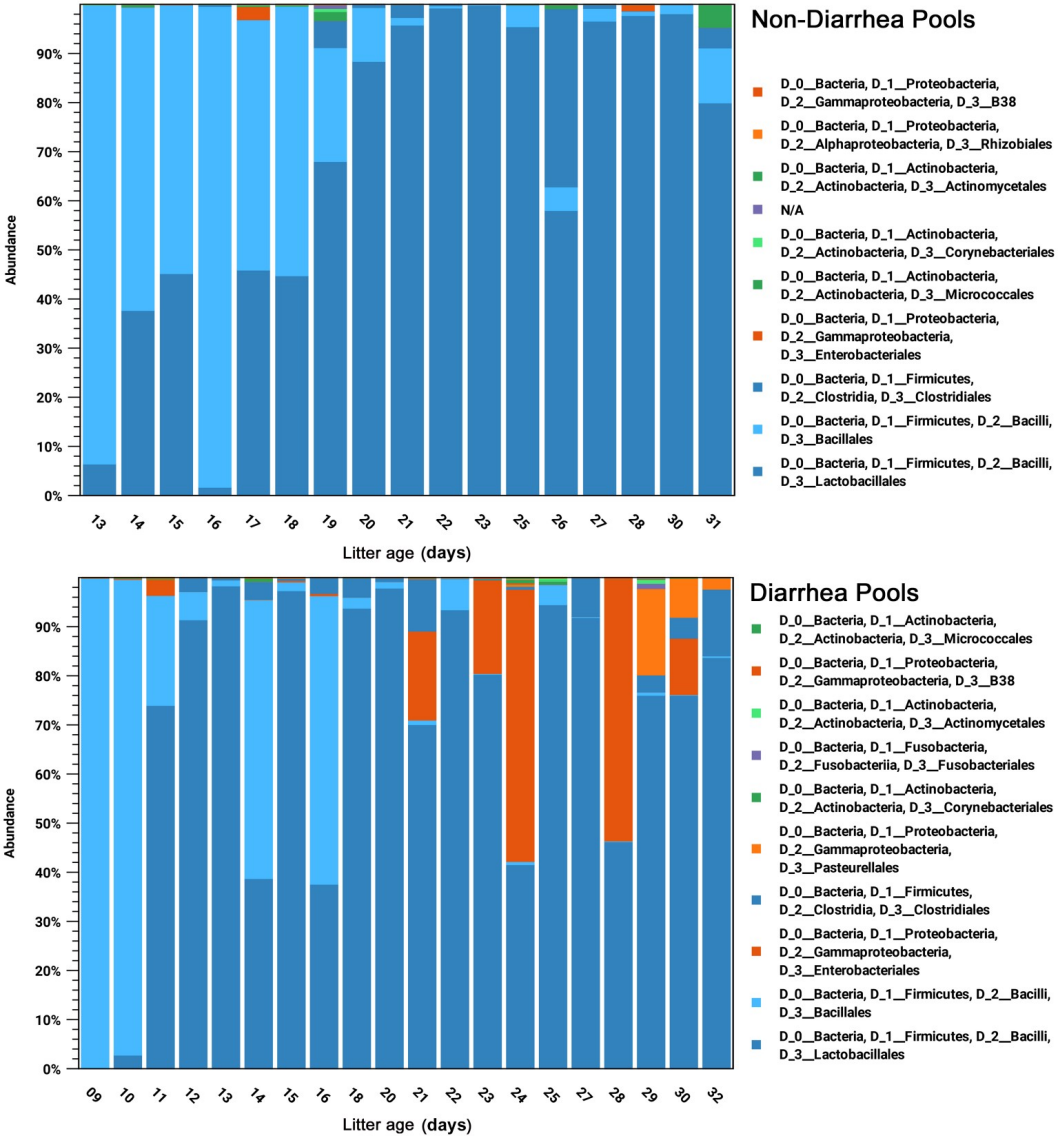
The bacterial populations of the 83 mink kit feces pool samples derived from 16S rRNA classification and grouped according to the litter age. Pools were grouped according the non-diarrheic (**top panel**) diarrheic (**bottom panel**) status. Populations are displayed aggregated and colored on the taxonomic order and phylum levels, respectively.

A detailed view of the same data displaying individual pools is show in Fig 2. In this figure the pools are grouped according to diarrheic and non-diarrheic. In addition the pools are ordered according to the corresponding litter age. For some of the litters the female had been treated with amoxicillin and the corresponding pools have been indicated with a star (*). It is seen that there is complete dominance by Bacillales and Lactobacillales among the non-diarrheic pools with more than 80% of the assigned amplicons. On the other hand, among the diarrheic pools there is a clear tendency for more significant contributions of Proteobacteria to the bacterial flora mainly constituting of *Enterobacter*, *Escherichia-Shigella* and Clostridia (not shown on this taxonomic level). Also a single diarrheic pool (no 82) with the bacterial population dominated by *Pasteurella* species is seen. From Fig 1 it is also clear that the enterococci increased in the diarrheic group accounting for 70% of the reads as compared to 50% in the non-diarrheic group. This increase was at the expense of staphylococci and streptococci that together accounted for almost 45% of the reads in the non-diarrheic group but only 17% in the diarrheic group (Fig 1). From Fig 2, it can be seen that this general pattern emanates from a gradual appearance and dominance by Proteobacteria with increasing litter age (c.f. Fig 3). The antibiotic treatment of the females does not seem to correlate with the composition of the microbiota in any way.

In Fig 3, the pools are ordered with respect to the litter age in days. Two trends are clearly apparent. The younger litters are dominated by Bacillales while Lactobacillales increase and become dominating with age. This is largely due to an increase of the enterococci and decrease of the staphylococci (c.f. Fig 1). In addition, for the diarrheic pools, there is a tendency that Proteobacteria become more prominent with age (c.f. Fig 1).

**Fig 4:**
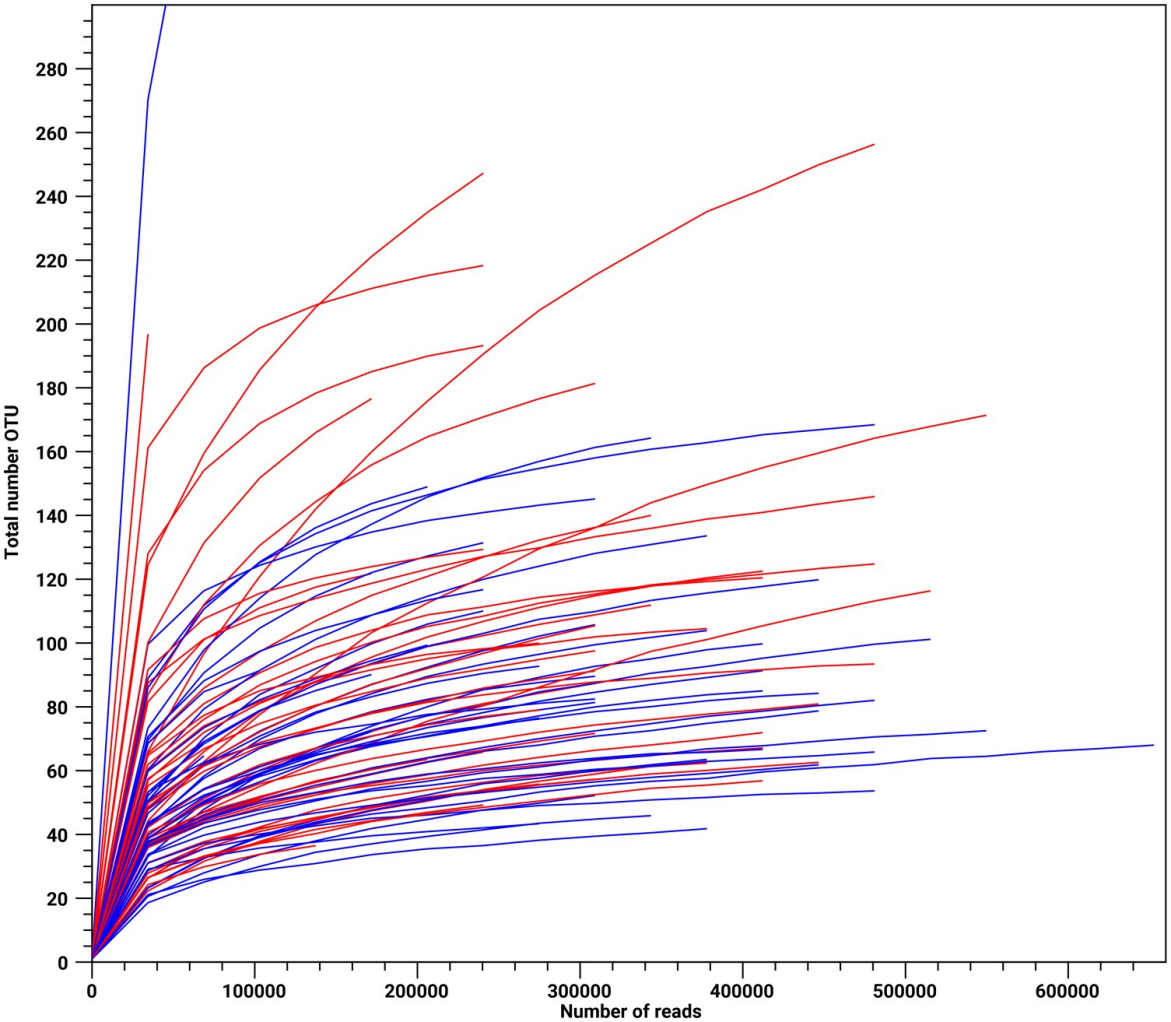
The α-diversity of the bacterial populations of the 83 mink kit feces pool samples displayed as the total number of operational taxonomic units at different numbers of sequence reads. Sampling was carried out without replacement and with 100 replicates. Pools classified as non-diarrheic and diarrheic are shown as blue and red curves, respectively.

**Fig 5:**
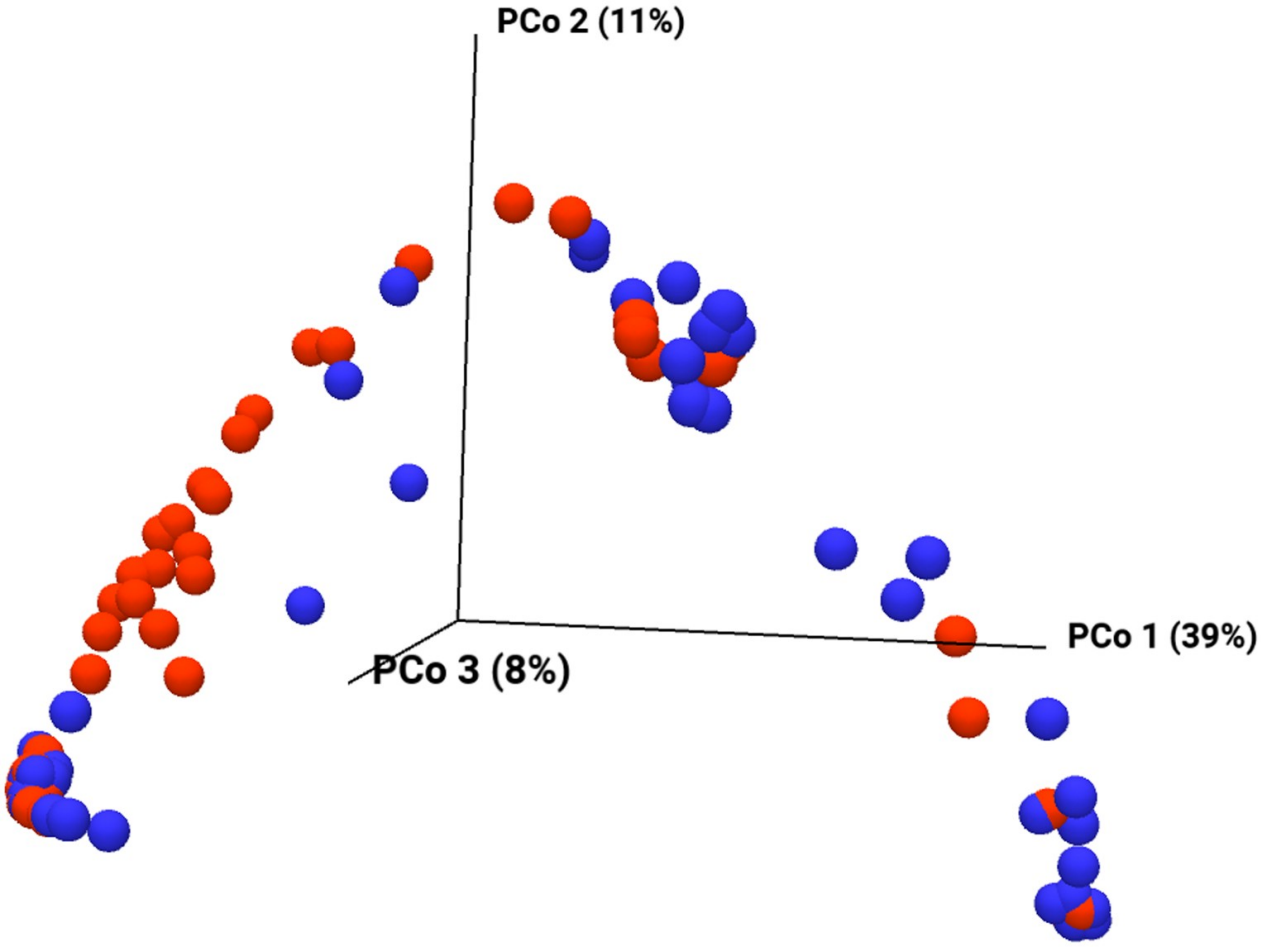
The β-diversity of the bacterial populations of the 83 mink kit feces pool samples. The β-diversity is shown as a three-dimensional principal component plot using the Jaccard diversity measure. Pools classified as non-diarrheic and diarrheic are shown as blue and red spheres, respectively.

The alpha diversity illustrates the species diversity at a habitat or a site; in our case a litter. The degree of species differentiation between habitats (i.e. between the different litters) is the beta-diversity [37]. In Fig 4 the alpha-diversity is illustrated by displaying the number of species as a function of the number of reads for each pool. The non-diarrheic samples are shown in blue while the diarrheic samples are shown in red. Overall, the diversity appears to be similar. A slight tendency for higher diversity in the diarrheic pools might be discernible (more red curves at the top of the graph). The beta diversity is shown in Fig 5 in a three-dimensional principal component plot. The non-diarrheic pools are shown in blue while the diarrheic pools are in red. The two groups do not form distinct clusters but have a clear tendency to separate mainly along the Pco1-axis. This is in accordance with the population results illustrated in Figs 1–3 that overall gives a clear picture that Proteobacteria becomes more predominant in diarrheic pools than the non-diarreic pools.

### Virome

**Fig 6:**
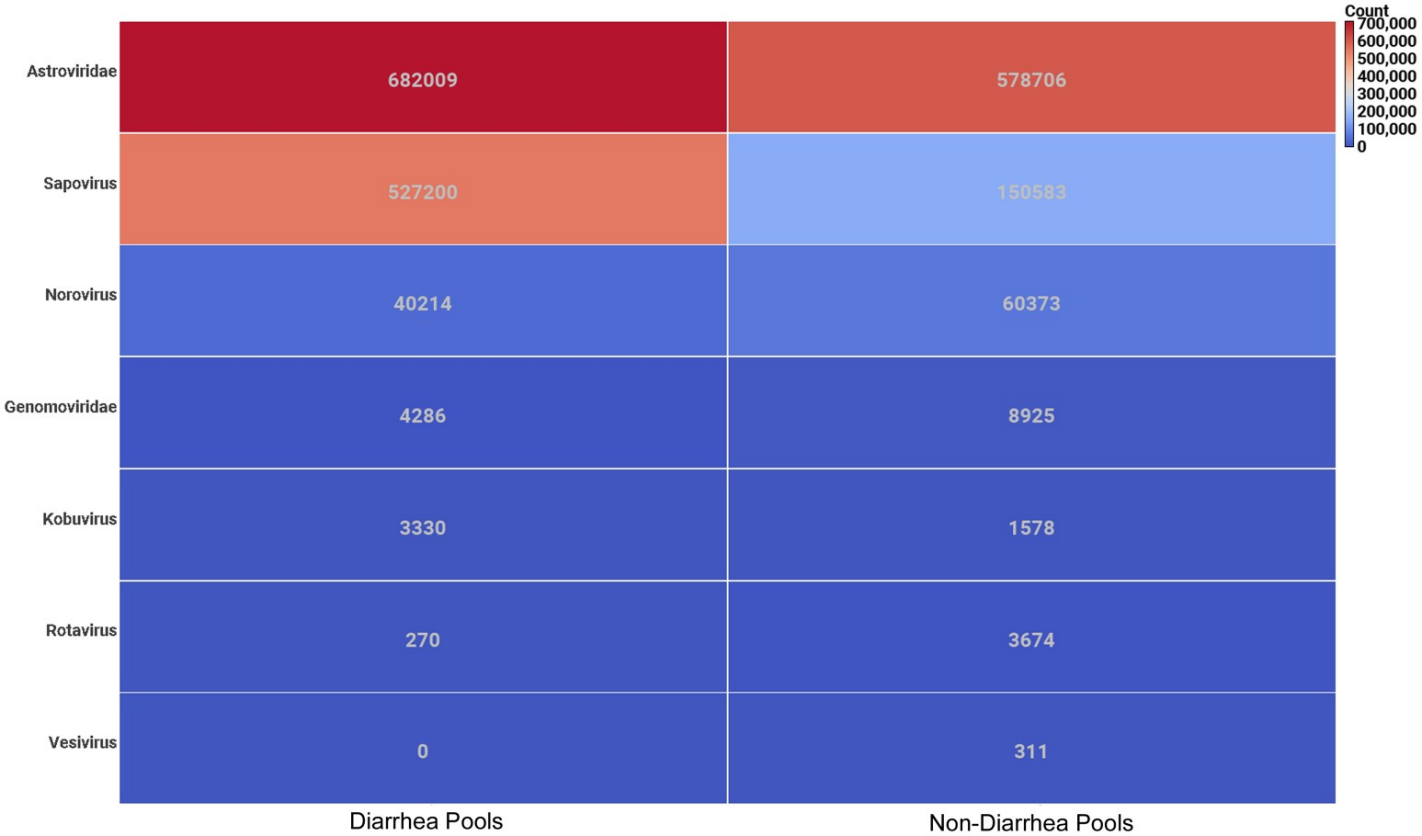
The number of sequence reads assigned to viruses for 61 mink kit feces pool samples. Of the 83 mink kit feces pool samples 61 (30 diarrheic and 31 non-diarrheic) produced sufficient data (more than 95 000 reads) for comparative study. Pools classified as non-diarrheic and diarrheic are shown to the left and right, respectively. A cut-off has been employed where viruses with less than 1000 reads assigned are not shown except vesiviruses that belong to the *Caliciviridae* family.

The 300 nt paired-end sequencing reads acquired were taxonomically categorized and analyzed in the diarrheic and non-diarrheic groups (see Material and methods for details). In Fig 6 the viral content in terms of number of reads assigned to different virus groups are shown for diarrheic and non-diarrheic pools in terms of a heat map with reddish and bluish colors corresponding to many and few assigned reads, respectively. A cut-off limit of 1000 reads has been employed. In addition, viruses infecting bacteria have been excluded. Vesiviruses, lagoviruses, sapoviruses and noroviruses all belong to the *Caliciviridae* family. Caliciviruses and astroviruses were prevalent in both diarrheic and non-diarrheic pool samples and they were found in large amounts (Fig 6). The number of reads assigned to caliciviruses was almost three times as many in the diarrheic group as in the non-diarrheic group of pools. *Sapporo*-like caliciviruses dominated and were almost ten times more abundant than *Norwalk*-like caliciviruses. Furthermore, the sapoviruses were even more dominating in the diarrheic pools since the noroviruses actually were more prevalent in the non-diarrheic pools (Fig 6). The amount of reads assigned to astroviruses was even more abundant than the caliciviruses but was more similar between the two groups with 10% more reads among the diarrheic pools. Astroviruses were of the mamastrovirus genus and almost exclusively of type 10 as usually found in mink. Kobuviruses and rotaviruses were quite common in the pools but usually occurred in relatively small quantities that overall did not differ much between diarrheic and non-diarrheic pools. Other viruses occurred sporadically and in small quantities.

## Discussion

The application of HTS technologies to characterize the virome in diarrheic and healthy mink kits showed that mink kits host many different viruses in their gut some of which also have been found in adult mink and ferrets [38–39]. The most prevalent eukaryotic viruses were caliciviruses (*Norovirus* and *Sapovirus*) and astroviruses. As expected from several previous studies caliciviruses and astroviruses were prevalent in both diarrheic and non-diarrheic samples [10–12,14]. *Kobuvirus* and rotavirus were also detected but with smaller number of reads. Calicivirus was found in higher amounts in pools from mink kits with diarrhea compared to non-diarrheic mink kits which is consistent with an earlier study which found calicivirus to be associated with PWD [10]. Furthermore, *Sapporo*-like viruses (SLV, Sapovirus) dominated in the pools supporting the results from a previous study where mink enteric calicivirus (MEC) was characterized as being close related to SLV viruses in other animal species [13]. However, caliciviruses were also found in considerable amounts in pools from healthy kits which corresponds to results from previous work [10,14]. The number of reads assigned to astrovirus was not significantly different between diarrheic and non-diarrheic pools. This contrasts the discovery, that astrovirus was found to be significantly associated with PWD both on farm and kit level [10]. This discrepancy might be explained by the complexity of multifactorial diseases where incubation period, infective dose, immune competence and co-infections with other pathogens might influence the outbreak of the syndrome. There might also be strains with different pathogenic potential since a huge diversity of astroviruses has been found in other species, including human [40–42]. We might also have expected a difference if non-diarrheic pools had solely been sampled from farms without PWD at all, but in fact, that was not the situation with the present material from 2015 where most of the included farms experienced problems with PWD to some extent. Astrovirus have been found in adult healthy mink and ferrets [38–39] as well as in a wide range of animal species primary affecting young individuals where they appear to have acquired host-specificity [43–44]. In humans e.g., astrovirus symptoms of infection range from unapparent or very mild signs of diarrhea to less commonly vomiting, fever and even mortality in children, elderly and immune compromised people [45–47]. Thus, based on a general behavior of astrovirus, it is not unlikely that mink kits can host astrovirus without expressing obvious signs of diarrhea.

Our study groups were suckling mink kits which had not been introduced to solid feed, and therefore their composition of gut bacteria was expected to be not so diverse and to differ from adult minks since the development of the bacterial microbiota is dependent on age and type of feeding [23,25]. Our results showed that the major groups of bacteria in both healthy and diseased animals were enterococci and staphylococci which differ from the composition in adult mink where Clostridia, Fusobacteria and Gammaproteobacteria dominate [27–29,48]. Large differences between the pools were seen and seem to correlate with litter age (Figs 2 and 3). This is consistent with results from another study which noted large inter-individual variation of the bacterial flora of adult mink [27]. The present study showed that Bacillales (staphylococci) were dominating in the youngest mink kits and were gradually outnumbered by Lactobacillales (enterococci) (Fig 3) which is supported by a previous culture based study [24]. The *Staphylococcus* group was decreased in the diarrheic pools compared to the non-diarrheic pools which indicates that this group of bacteria is not likely involved in the etiology of PWD syndrome but supports previous findings that staphylococci (*Staphylococcus delphini*) are natural inhabitants in mink [20,23–24]. In diarrheic pools, we found a clear tendency for more contributions to the bacterial flora of *Enterobacter*, *Escherichia-Shigella* and Clostridia, which emerged at the expense of not only staphylococci but also streptococci. *E*.*coli* has been studied in both healthy and diarrheic mink kits but it has not been possible to identify certain types of *E*.*coli* involved in the pre-weaning diarrhea syndrome [14,18]. The pronounced increase in the number of reads assigned to enterococci in the diarrheic group raises questions about their pathogenic potential, and in e.g. suckling rats, piglets and kittens enterococci have been associated with diarrhea [49–53]. Interestingly, the combination of increased proportion of enterococci and *E*.*coli* that we found in the diarrheic group also has been demonstrated in piglets with New Neonatal Porcine Diarrhea (NNPD) [32,52,54], suggesting some similarities in pathology between mink kits and piglets with diarrhea in the suckling period. Clostridia and *Escherichia-Shigella* are major components of the adult bacterial microbiota [27,29], and based on these findings PWD in mink kits may be interpreted as a general result of less tolerance of too early colonization by specific bacteria groups. Material for this study was collected from commercial mink farms with severe on-going outbreaks of PWD and therefore it was impossible to control and avoid antimicrobial treatment of the females in all the sampled litters. However, none of the sampled mink kits were treated and from Fig 2, this does not seem to influence the composition of the mink kit microbiota and the overall results.

In the present study, we did not see a clear tendency that the diversity of the bacterial community is strongly influenced (Fig 5) but the relative amounts of different bacteria were affected. Similarily, the viruses detected overall appeared both in diarrheic and non-diarrheic pools but the amounts differed significantly in particular for sapoviruses. In conclusion, this study has, with application of NGS methods, provided information about the bacterial and viral community in healthy and diarrheic mink kits. Thus, our data support a model where there is no single etiological agent but there is a balance in the microbiota that is perturbed in the diarrheic animals where both caliciviruses and astroviruses increase and simultaneously enterococci, *Enterobacter* and Clostridia become more prevalent at the expense of staphylococci and streptococci. Possibly, there could be certain variants of caliciviruses and astroviruses that are more pathogenic and play a leading role in this scenario which is currently under investigation. Further investigation with more specific methods is needed to clarify the interplay between certain agents and the host in the pre-weaning diarrhea syndrome of mink.

## Supporting information

S1 Fig Scoring of rectal contents/feces in mink kits. The numbers refer to the consistency of the feces and the letter to the color. A: Score 1; Firm to normal soft, log-shaped and moist with smooth surface. B: Score 2; Soft without shape, very moist, cow-pat like consistency. C: Score 3; Runny, loose, no defined shape with some texture. Also notice external signs: a sticky exudation on the skin, red swollen anus and black claws. D: Score 4; Liquid, not containing any particular matter, no texture and may be foamy. E: Score a; Undigested, white or beige color. F: A mink litter affected with PWD and cutaneous exudation located to the neck, legs and paws. Unpublished data submitted to Acta Vet Scand.
(TIF)

S1 Table Definition of disease status. Unpublished data submitted to Acta Vet Scand.
(PDF)
