# Supplementary figures and images for: Investigation of the viral and bacterial microbiota in intestinal samples from mink (Neovison vison) with pre-weaning diarrhea syndrome using next generation sequencing
Source: PLoS One. 2018 Oct 18;13(10):e0205890. doi: 10.1371/journal.pone.0205890 (PMC6193705; doi:10.1371/journal.pone.0205890)

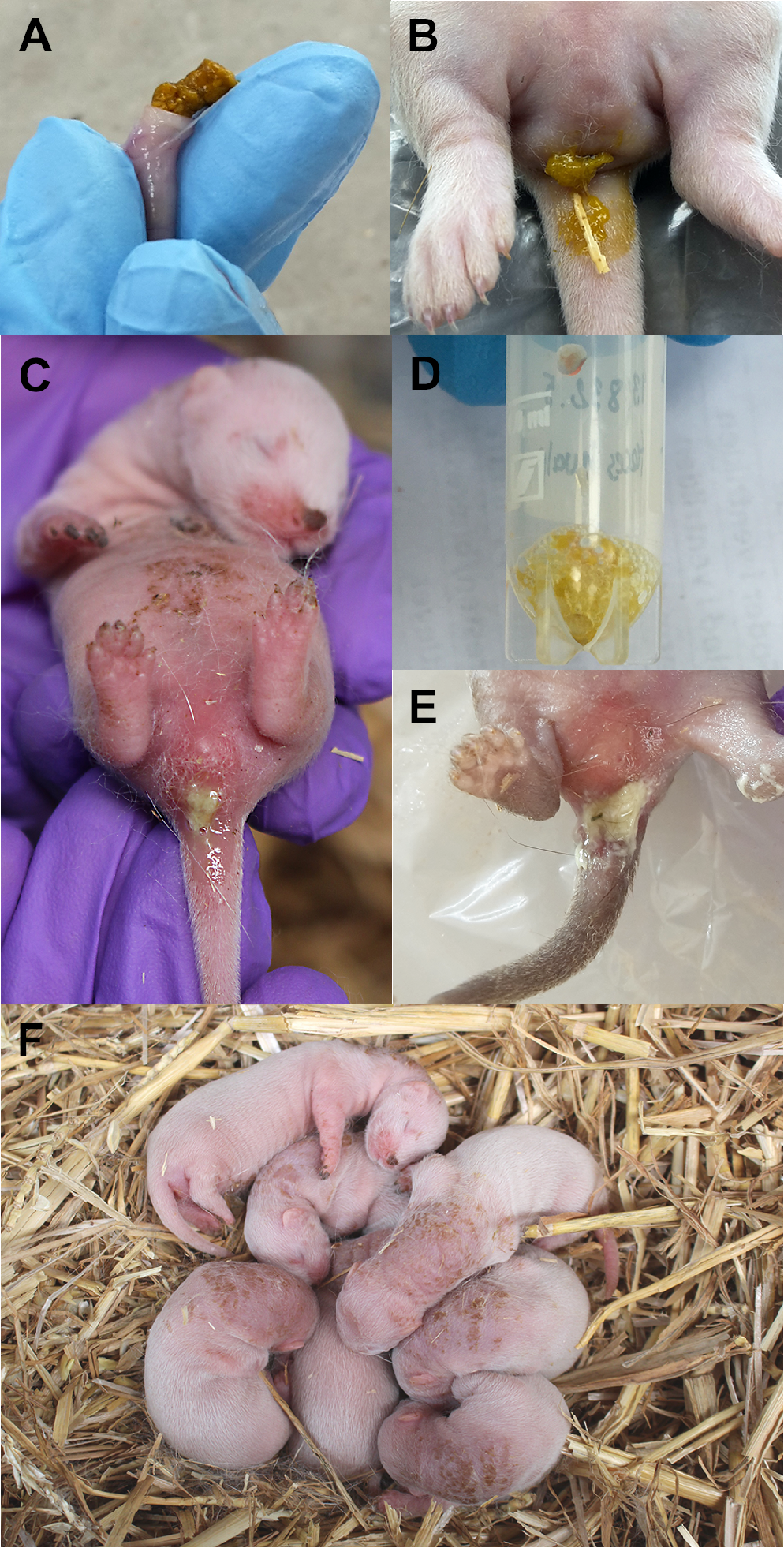

Supplement: S1 Fig — The numbers refer to the consistency of the feces and the letter to the color. A: Score 1; Firm to normal soft, log-shaped and moist with smooth surface. B: Score 2; Soft without shape, very moist, cow-pat like consistency. C: Score 3; Runny, loose, no defined shape with some texture. Also notice external signs: a sticky exudation on the skin, red swollen anus and black claws. D: Score 4; Liquid, not containing any particular matter, no texture and may be foamy. E: Score a; Undigested, white or beige color. F: A mink litter affected with PWD and cutaneous exudation located to the neck, legs and paws. Unpublished data submitted to Acta Vet Scand. (TIF) [file pone.0205890.s001.tif]
