# Supplementary material for: Investigation of the viral and bacterial microbiota in intestinal samples from mink (Neovison vison) with pre-weaning diarrhea syndrome using next generation sequencing
Source: PLoS One. 2018 Oct 18;13(10):e0205890. doi: 10.1371/journal.pone.0205890 (PMC6193705; doi:10.1371/journal.pone.0205890)
Supplement: S1 Table — Unpublished data submitted to Acta Vet Scand. (PDF) [file pone.0205890.s002.pdf]

Pre-weaning diarrhea mink kit	
Feces score*	if score 3 OR<br>if score 4 OR<br>if score a
If empty rectum or no feces to evaluate OR score 2 (cow-pat like feces)	if swollen or red anus OR<br><br>if dirty perineal region OR<br><br>if cutaneous exudation on head, leg, trunk, tail or paws

Healthy mink kit	
Feces score*	if score 1 OR<br>if score 2<br><br>AND
External assessment	no red or swollen anus AND<br><br>no dirty perineal region AND<br><br>no cutaneous exudation on head, legs, trunk tail or paws
If empty rectum or no feces to evaluate	If no red or swollen anus AND<br><br>no dirty perineal region AND<br><br>no cutaneous exudation on the head, legs, trunk, tail, or paws<br><br>AND litter mates have score 1

*Evaluated in rectum or from post mortem defecation

Unpublished data submitted to Acta Vet Scand.
